# Identification of Amino Acids that Account for Long-Range Interactions in Two Triosephosphate Isomerases from Pathogenic Trypanosomes

**DOI:** 10.1371/journal.pone.0018791

**Published:** 2011-04-18

**Authors:** Itzhel García-Torres, Nallely Cabrera, Alfredo Torres-Larios, Mónica Rodríguez-Bolaños, Selma Díaz-Mazariegos, Armando Gómez-Puyou, Ruy Perez-Montfort

**Affiliations:** Departamento de Bioquímica y Biología Estructural, Instituto de Fisiología Celular, Universidad Nacional Autónoma de México, Circuito Exterior S/N, Ciudad Universitaria, México DF, Mexico; University of Hong Kong, Hong Kong

## Abstract

For a better comprehension of the structure-function relationship in proteins it is necessary to identify the amino acids that are relevant for measurable protein functions. Because of the numerous contacts that amino acids establish within proteins and the cooperative nature of their interactions, it is difficult to achieve this goal. Thus, the study of protein-ligand interactions is usually focused on local environmental structural differences. Here, using a pair of triosephosphate isomerase enzymes with extremely high homology from two different organisms, we demonstrate that the control of a seventy-fold difference in reactivity of the interface cysteine is located in several amino acids from two structurally unrelated regions that do not contact the cysteine sensitive to the sulfhydryl reagent methylmethane sulfonate, nor the residues in its immediate vicinity. The change in reactivity is due to an increase in the apparent pKa of the interface cysteine produced by the mutated residues. Our work, which involved grafting systematically portions of one protein into the other protein, revealed unsuspected and multisite long-range interactions that modulate the properties of the interface cysteines and has general implications for future studies on protein structure-function relationships.

## Introduction

It is assumed that the structure-function relationship from similar protein sequences will usually yield similar physicochemical and functional properties. Take for example the glycolytic enzyme triosephosphate isomerase (TIM) from two evolutionarily related pathogenic parasites, Trypanosoma brucei and T. cruzi. These are two pathogenic protists of the order of the kinetoplastidae that cause sleeping sickness and Chagas disease in humans, respectively. Many of the proteins of these parasites have a high degree of sequence identity; in the case of the two trypanosomal TIMs it is 73.9%, with a sequence similarity of 92.4%. Both enzymes are homodimers whose three dimensional structures superpose with an RMS of 0.96 Å and both have an identical catalytic site in each monomer formed by residues K13, H95 and E167 (based on the numbering for the sequence of TIM from *T. brucei* (TbTIM)).

However, even though the two enzymes are markedly similar, there are several striking differences in several functional properties of the two proteins. For example, their susceptibility to digestion with subtilisin [1], their velocity and extent of reactivation from guanidine chloride unfolded monomers [2], and their susceptibility to inactivation by several low molecular weight agents [3]. Of particular relevance to this work is their remarkably different susceptibility to sulfhydryl reagents like methylmethane thiosulfonate (MMTS): the enzyme from T. cruzi is 70 times more sensitive than the enzyme from T. brucei [4]–[8]. The initial site of action of the thiol reagent in both enzymes is their only interface cysteine (Cys), which is at position 14 or 15 of TbTIM and TIM from T. cruzi (TcTIM), respectively; it is surrounded by residues of loop 3 of the other subunit [4]–[11]. Since the three dimensional arrangements of the interface Cys relative to the other monomer are nearly identical in the two enzymes, the question arose as to which residues or parts of the enzymes are responsible for the different susceptibility to the thiol reagent.

The question of finding the amino acids in a protein sequence that have an influence on certain measurable protein functions has occupied protein chemists for many decades and, in consequence, numerous methods have been used to solve the problem [12]–[17]. Among the approaches to understand the relation between the structure and function of proteins, the use of chimeras has been rather frequent. Indeed, chimeras formed with different protein domains have been successfully used to ascertain the interplay between different portions of the protein and how each domain contributes to the overall function of the protein [18]–[20].

In this work, we show that by progressive grafting of different portions of a protein into equivalent regions of a homologous protein with a different trait, it is possible to ascertain the parts (and the amino acids) of the protein that participate in the expression of that feature.

A priori, we expected to find the determinants responsible for the susceptibility/resistance of the enzymes to MMTS either among the residues surrounding the interface Cys, or in residues that form the dimer interface, or in residues distributed throughout the whole protein. Instead, we found that the change in susceptibility to MMTS was due to residues that are not in contact with the interface Cys, and most are not part of the dimer interface, but belong to two specific regions of the protein that are not connected to each other either in a sequential or a structural basis. Our findings show that the assignment of a function or property to a few, or even a single amino acid, in functional and structural studies of proteins, should often be reconsidered and extended beyond the identification and definition of simple cavities or local interaction sites.

## Results

### Triosephosphate isomerase was divided into eight interchangeable modular regions

**Figure 1:**
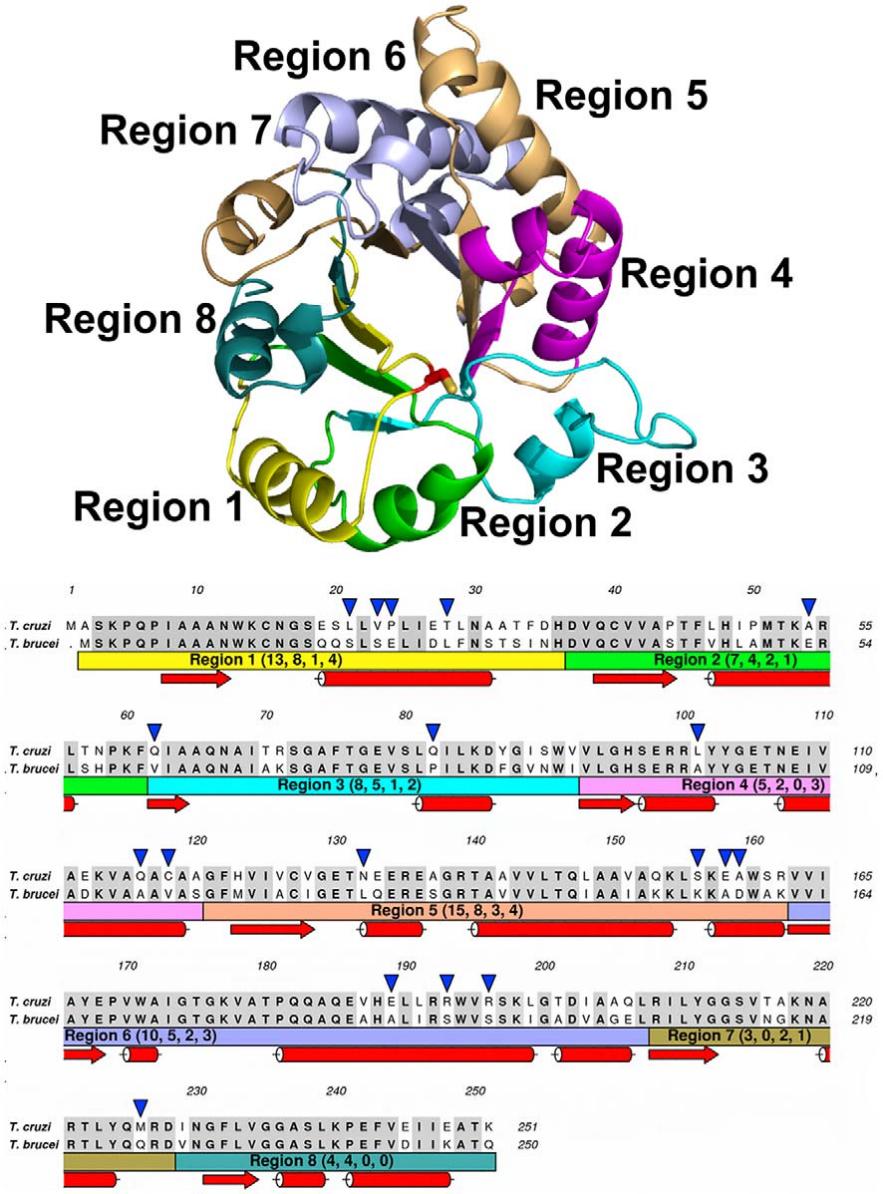
Position of the regions in the structure and aligned sequences of TbTIM and TcTIM. The ribbon diagram shows a monomer of TbTIM with each region in a different color and the interface Cys represented as sticks. The alignment of both sequences shows the identical amino acids shaded in grey. The color bars below indicate the region with the corresponding number of total differences, conserved substitutions, semiconserved substitutions and substitutions without homology in parenthesis, respectively. The blue arrowheads point to all substitutions without homology. Secondary structure elements of the sequences are also shown as red arrows (beta sheets) or cylinders (alpha helixes). The numbering of the amino acid sequence of TcTIM was used and the number and type of substitutions was taken from an alignment made using the Clustal W algorithm.

Because of its octamerous β/α barrel fold, the sequence of both TIMs was divided into eight regions that approximately correspond to a beta sheet, the corresponding beta-alpha loop and the alpha helix (Figure 1). In order to determine the amino acids that account for the different susceptibility of the two enzymes to MMTS, we gradually transformed TcTIM into TbTIM by creating chimeras that had an increasing number of TbTIM regions and examined their susceptibility to the action of MMTS. Figure 1 depicts the amino acids that comprise each region and the differences in their amino acid sequence. There are 65 different residues in the two enzymes, the majority of them being in regions 1–6. We initially constructed six chimeras that contained a progressive number of TbTIM regions and a diminishing number of TcTIM regions; they were named according to their content of TcTIM regions. For example, the first chimera that was constructed is termed TcTIM 1–6 (it contains regions 1 to 6 of TcTIM and regions 7 and 8 of TbTIM), as a further example, we also constructed TcTIM 1 (it contains region 1 of TcTIM and regions 2–8 of TbTIM). Table 1 shows the chimeras used in this work.

**Table 1:** Method of purification used for wild type TbTIM, wild type TcTIM and each mutant enzyme and their kinetic constants.

Enzyme	Method of purification	*K*m (mM)	*k*cat ⋅10^5^ (min^−1^)	*k*cat/*K*m ⋅ 10^7^(M^−1^ s^−1^)
TbTIM	**300 mM NaCl**	0.45	3.10	1.15
TcTIM	**No NaCl**	0.43	2.70	1.05
TcTIM 1–6	**No NaCl**	0.13	0.96	1.23
TcTIM 1–5	**No NaCl**	0.38	1.55	0.68
TcTIM 1–4	**300 mM NaCl**	0.63	3.32	0.87
TcTIM 1–3	**300 mM NaCl**	0.58	3.31	0.95
TcTIM 1–2	**300 mM NaCl**	0.57	3.28	0.95
TcTIM 1	**300 mM NaCl**	0.48	3.17	1.10
TcTIM 4	**300 mM NaCl**	0.37	2.38	1.07
TcTIM 1–3, 5–8	**No NaCl**	0.44	2.60	0.97
TcTIM 2,3, 5–8	**No NaCl**	0.26	3.59	2.29

### Purification of the chimerical proteins

All the chimerical proteins were cloned and expressed in Escherichia coli. Different purification methods are used for wild type (WT) TbTIM and WT TcTIM, this is because after disruption of the cells and centrifugation, WT TcTIM partitions to the soluble fraction, whereas TbTIM localizes in the precipitate. Therefore, TbTIM was purified by treating the cell lysate with a 300 mM NaCl solution to solubilize the enzyme (see Methods). Due to these differences, in all the chimeras, the supernatant and precipitate obtained after cell disruption were analyzed by SDS-PAGE in order to ascertain whether the enzyme distributed in the soluble or insoluble fraction. According to the data, the corresponding chimera was purified by the procedures described for either TcTIM or TbTIM. Table 1 shows the purification method used for each chimera; in general, the chimeras that contained a majority of regions from TcTIM were purified from the soluble fraction, while those with more regions of TbTIM were purified by adding 300 mM NaCl to the lysis buffer (see Materials and Methods). The yield of pure protein for the chimeras was similar to that of the WT enzymes (60–80 mg/L of culture). Only chimera TcTIM 1 yielded lower quantities of purified protein (approximately 25 mg/L of culture).

### The catalytic properties of the chimerical proteins are similar to those of the wild-type enzymes

The steady state kinetics of all chimerical enzymes were determined in the direction of glyceraldehyde 3-phosphate to dihydroxyacetone-phosphate. The Km and kcat values of the chimeras and WT enzymes were within the same range (Table 1). The kinetic parameters of chimera TcTIM 1–6 were lower when compared with the WT enzymes; nevertheless, its catalytic efficiency (kcat/Km) was similar to that of the WT enzymes. The catalytic efficiency of TcTIM 1–5 was approximately one half of that of the WT TIMs, mainly due to a lower kcat.

### The non-identical amino acids of regions 5 to 8 (49%) are not involved in MMTS susceptibility

**Figure 2:**
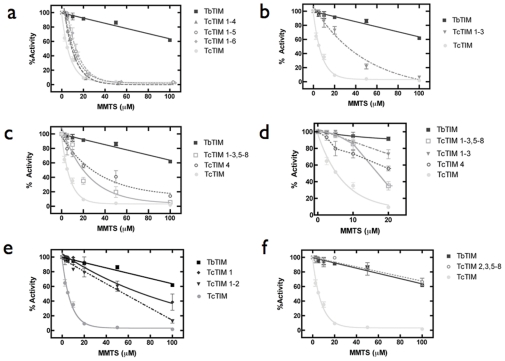
Effect of MMTS on WT TcTIM, WT TbTIM and on different chimeras. The enzymes were incubated at a concentration of 250 µg/mL in 100 mM TEA, 10 mM EDTA, and the indicated concentrations of MMTS (pH 7.4) for 2 h. At that time the activity of the samples was determined, including a sample without MMTS to calculate the percentage of remaining activity. Panel a) Effect of MMTS on WT TcTIM, WT TbTIM and chimeras TcTIM 1–6, TcTIM 1–5 and TcTIM 1–4. Panel b) Effect of MMTS on WT TcTIM, WT TbTIM and on chimera TcTIM 1–3. Panel c) Effect of MMTS on WT TcTIM, WT TbTIM and on two chimeras of region 4: TcTIM 1–3, 5–8 and TcTIM 4. Panel d) Close up of the first part of the curves shown in panel c, including the data for chimera TcTIM 1–3 shown on Panel b. Panel e) Effect of MMTS on WT TcTIM, WT TbTIM and on chimeras TcTIM 1–2 and TcTIM 1. Panel f) Effect of MMTS on WT TcTIM, WT TbTIM and on chimera TcTIM 2,3, 5–8.

The sulfhydryl reagent methylmethane thiosulfonate (MMTS) inactivates TbTIM and TcTIM by reacting initially with their only interface Cys 14 or Cys 15, respectively [4]–[11]. Confirming previous results [21], we observed that the exposure of WT TcTIM and WT TbTIM to MMTS induced abolition of catalysis and that TcTIM was about 70 times more sensitive to the sulfhydryl reagent than TbTIM (Figure 2). Because the two enzymes are markedly similar in amino sequences and crystal structures, we sought to find which region or regions are responsible for the difference in susceptibility of TbTIM and TcTIM to the inactivating action of MMTS. Thus, we determined the effect of different concentrations of MMTS on the catalytic activity of the chimeras. The three chimeras TcTIM 1–6, TcTIM 1–5 and TcTIM 1–4 exhibited an inactivation pattern similar to that of WT TcTIM (Figure 2 Panel a). It is noteworthy that the susceptibility to MMTS of chimera TcTIM 1–4 that has one half the sequence of each of the two enzymes and a difference of 33 amino acids with TbTIM (87% identity) is similar to that of WT TcTIM.

### Region 4 is involved in the susceptibility of TcTIM and TbTIM to low MMTS concentrations

When region 4 of TbTIM was subsequently incorporated in chimera TcTIM 1–4 to produce chimera TcTIM 1–3, an important change in the inactivation pattern with MMTS was observed (Figure 2 Panel b). This chimerical enzyme was the first in which we observed a pattern of inactivation by MMTS that resembled that of TbTIM. A salient feature of the MMTS inhibition curve of this chimera is that similarly to WT TbTIM, it retains 100% activity at concentrations below 10 µM MMTS. These findings indicated that the five differences in the amino acid sequences in region 4 (Figure 1) contribute to the susceptibility of WT TcTIM and WT TbTIM to MMTS. For this reason, we made two new chimeras: a TbTIM that had only region 4 of TcTIM (TcTIM 4) and a TcTIM that had only region 4 of TbTIM (TcTIM 1–3, 5–8). The susceptibility to the inactivating effect of MMTS of these two chimeras is shown in Figure 2 Panel c. Remarkably, the chimeras showed an overall intermediate response between the susceptible WT TcTIM and the resistant WT TbTIM. Nevertheless, there is an important difference between them (Figure 2 Panel d); chimera TcTIM 4, with only region 4 of TcTIM, started to loose activity with 2.5 µM MMTS, in the same way as WT TcTIM. Conversely, chimera TcTIM 1–3, 5–8 was hardly affected by low concentrations of MMTS, resembling WT TbTIM. Thus, the five different amino acids in the sequences of region 4 (Figure 1) are active participants in the overall response of the WT enzymes to MMTS.

### Region 1 is instrumental in the susceptibility of TcTIM and TbTIM to high MMTS concentrations

Because chimeras with alternate regions 4 of TcTIM or TbTIM showed intermediate susceptibility to the inactivating action of MMTS, we tested two chimeras that contained additional regions of TbTIM: chimera TcTIM 1–2 and chimera TcTIM 1. The susceptibility of these chimeras to MMTS is shown in Figure 2 Panel e. TcTIM 1–2 retained most of its activity at concentrations below 20 µM MMTS; at higher concentrations, its activity was progressively inhibited and with 100 µM MMTS, inhibition was almost complete. A similar phenomenon occurred with chimera TcTIM 1, at low MMTS concentrations, it exhibited almost full activity; at higher concentrations, activity started to decrease, reaching 40% of its original activity with 100 µM MMTS. Thus, at high MMTS concentrations the behavior of TcTIM 1 approached, but still did not equal that of WT TbTIM.

The latter observations indicated that region 1 plays a central role in the susceptibility of the enzymes to MMTS; likewise the data of Figure 2 Panels c and d show that region 4 has a strong influence on the response to the thiol reagent. We therefore designed a chimera of TcTIM with regions 1 and 4 of TbTIM (TcTIM 2,3, 5–8). This chimera and WT TbTIM exhibited almost identical inactivation profiles at low and high concentrations of MMTS (Figure 2 Panel f). Thus, by using a region exchange method, we were able to build a chimera that had an MMTS inactivation profile undistinguishable from that of WT TbTIM. Taken together, the data with chimeras TcTIM 1 and TcTIM 2,3, 5–8 it may be concluded that, at most, the 13 different amino acids in region 1 of the two WT enzymes account for their different susceptibilities to MMTS at concentrations higher than 50 µM.

**Figure 3:**
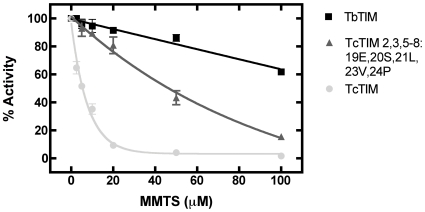
Effect of MMTS on WT TcTIM, WT TbTIM and on mutant TcTIM 2,3, 5–8: 19E, 20S, 21L 23V, 24P. The enzymes were incubated at a concentration of 250 µg/mL in 100 mM TEA, 10 mM EDTA, and the indicated concentrations of MMTS (pH 7.4) for 2 h. At that time the activity of the samples was determined, including a sample without MMTS to calculate the percentage of remaining activity.

Initial experimental proof that these 13 different amino acids are involved in this difference of behavior was obtained by mutational analysis of some of the residues. Using TcTIM 2,3, 5–8 as the template, we prepared mutant TcTIM 2,3, 5–8:19E, 20S, 21L, 23V, 24P (reverting the first five different amino acids in region 1 of TbTIM to those found in the sequence of TcTIM). The susceptibility of this mutant to MMTS was determined in the same conditions as those of the other chimeras and the result showed that it had an intermediate susceptibility between WT TbTIM and WT TcTIM (Figure 3). Other double and single mutants namely: TcTIM 2,3, 5–8:19E, 20S; TcTIM 2,3, 5–8: 21L, 23V and TcTIM 2,3, 5–8: 24P tended to have a susceptibility to MMTS which was more similar to the original template (supplementary Figure S1) indicating that these first five amino acids are largely responsible for the susceptibility of TcTIM to high concentrations of MMTS. The kinetic parameters of all these four mutants were very similar to those of TcTIM 2,3, 5–8 (supplementary Table S1).

Since TcTIM has a higher susceptibility than TbTIM towards other thiol reactive agents like 5,5-dithiobis(2-nitrobenzoate) (DTNB), 4,4 dithiopyridine, and n-ethylmaleimide [4], we also tested the inactivation of different chimeras with 1 mM DTNB. Table 2 shows that Cys14 of TbTIM needs 18 min to derivatize while Cys15 of TcTIM takes less than a minute. After twelve minutes, approximately 4 of the 8 Cys in a dimer of WT TcTIM are derivatized, while approximately one Cys per dimer from the 6 Cys in the dimer of TbTIM has reacted. As can be seen from the corresponding time at which the interface Cys was derivatized and the number of Cys derivatized in the dimers of the chimeras by DTNB at 12 minutes, the behavior of all proteins paralleled the inactivation scheme they had shown in the presence of MMTS, indicating that the susceptibility to this reagent is also affected by the amino acids in regions 1 and 4.

**Table 2:** Derivatization of Cys by DTNB in the dimers of wild type TbTIM, wild type TcTIM and nine mutant enzymes.

Enzyme	Cys per dimer	Time for derivatization of the first cysteine (Cys14 or 15)	Derivatized Cys per dimer after twelve minutes with DTNB
TbTIM	6	18 min	0.9
TcTIM	8	<1 min	3.8
TcTIM 1–6	8	<1 min	4.7
TcTIM 1–5	8	<1 min	4.7
TcTIM 1–4	8	<1 min	3.4
TcTIM 1–3	6	3 min	2.0
TcTIM 1–2	6	9 min	1.6
TcTIM 1	6	6 min	1.8
TcTIM 4	8	4 min	2.6
TcTIM 1–3,5–8	6	6 min	2.0
TcTIM 2,3, 5–8	6	>20 min	0.33

### The interface Cys does not contact the residues of regions 1 and 4 that confer different susceptibility to MMTS

**Figure 4:**
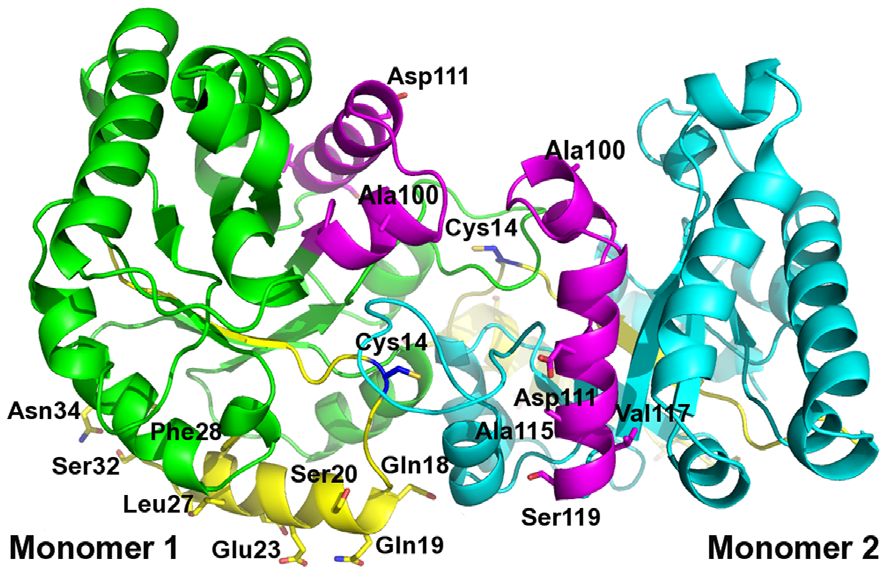
Three-dimensional localization of regions 1 and 4. Representation of the TIM dimer and the 18 residues that differ between region 1 and 4 of TbTIM and TcTIM. The color code used for regions 1 and 4 is the same as in Figure 1, and the two monomers are colored in green and turquoise. The altered residues are highlighted as sticks. Sequence numbers are according to TbTIM (PDB code 5TIM), and register as 1 amino acid less, when compared to TcTIM.

The three dimensional location of the residues in regions 1 and 4 that account for the different susceptibility to MMTS in TcTIM and TbTIM shows several surprising features (Figure 4). None of the 18 residues that are relevant for the susceptibility of the interface Cys are in contact with this residue, nor with those that surround it. Also, according to an analysis of the structure of TbTIM (PDB code 5TIM) with the PISA server [22], only one of the 18 residues in regions 1 and 4 forms part of the dimer interface, albeit in the case of TcTIM (PDB code 1TCD), 18Q–19E may be considered part of the interface with a buried surface area of 64 Å^2^. Finally, there are no contacts between the relevant residues of regions 1 and 4 (Figure 4). Thus, from the structural point of view, it would seem that the effects of region 1 are independent from those of region 4, and vice versa. Altogether, the data show that the relevant residues of regions 1 and 4 modulate the reactivity of the interface Cys through long-range interactions and that the interface residues do not play a role in the susceptibility to MMTS.

**Figure 5:**
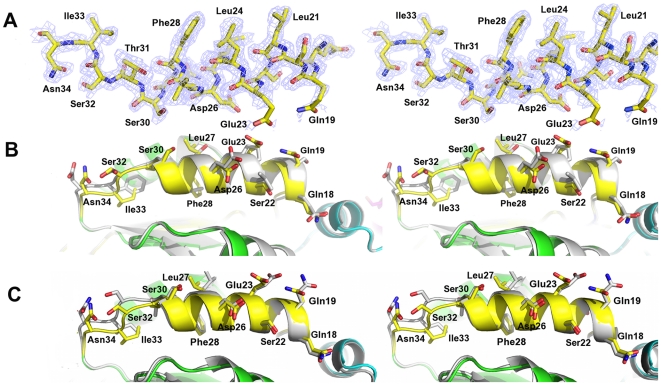
Region 1 of the crystal structure of chimera TcTIM 2,3, 5–8. A. Stereo view of sigma weighted, 2Fo-Fc simulated annealing omit map contoured at 1σ for region 1 in the final model of the crystal structure of chimera TcTIM 2,3, 5–8 (colored ribbon). B. Superposition on TcTIM (grey cartoon). The rmsd value for this region is 0.363 Å, for the Cα atoms. C. Superposition on TbTIM (grey cartoon). The rmsd value for this region is 0.237 Å. The sequence numbers are the same as in Figure 4.

**Figure 6:**
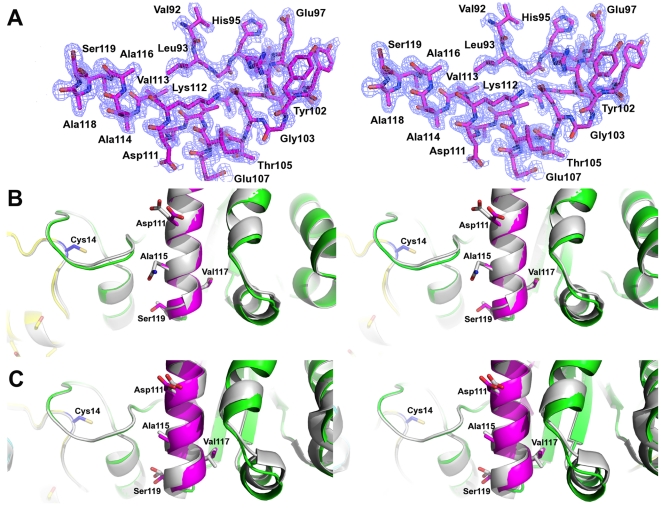
Region 4 of the crystal structure of chimera TcTIM 2,3, 5–8. A. Stereo view of sigma weighted, 2Fo-Fc simulated annealing omit map contoured at 1σ for region 4 in the final model of the crystal structure of chimera TcTIM 2,3, 5–8 (colored ribbon). B. Superposition on TcTIM (grey cartoon). The rmsd value for this region is 0.219 Å, for the Cα atoms. C. Superposition on TbTIM (grey cartoon). The rmsd value for this region is 0.150 Å. The sequence numbers are the same as in Figure 4. The interface Cys is shown as blue sticks.

**Figure 7:**
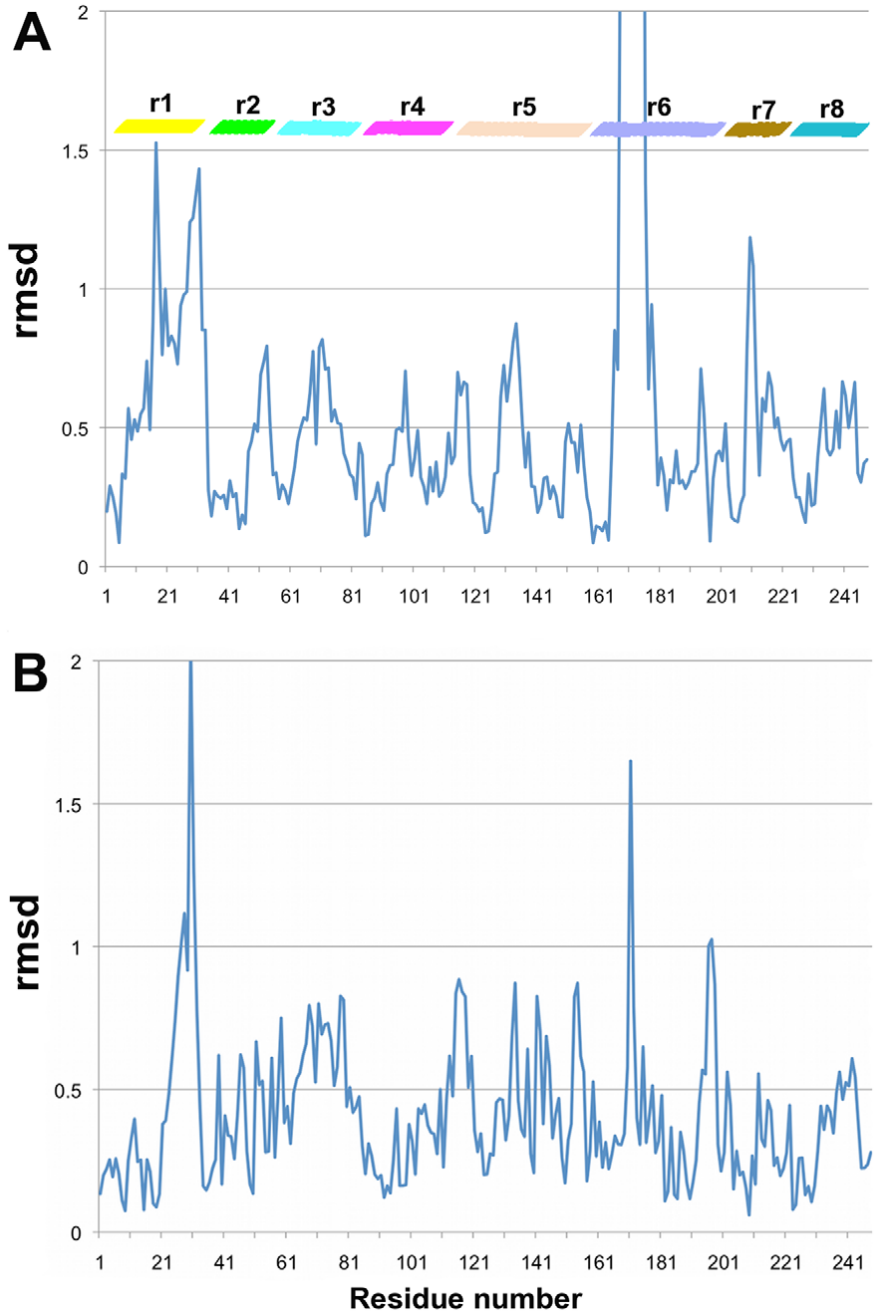
Root mean square deviations between the structures of chimera TcTIM 2,3,5–8, TcTIM and TbTIM. A. RMS deviations between chimera TcTIM 2,3, 5–8 and TcTIM (PDB code 1TCD), overall rmsd 0.385 Å. B. RMS deviations between chimera TcTIM 2,3, 5–8 and TbTIM (PDB code 5TIM), overall rmsd 0.437 Å. The relative location of the 8 TIM regions is indicated in color bars using the same color code as in Figure 1.

In order to check for the absence of major conformational differences due to the sequence perturbations on the chimeras, we solved the crystal structure at 1.65 Å resolution of chimera TcTIM 2,3, 5–8 (Figs. 5 and 6). The analysis of the structure shows that the chimera can be superposed on the crystal structures of TcTIM and TbTIM with a RMSD of 0.385 and 0.437 Å respectively, with a minor displacement on the loop of region 1 (Figure 7). A displacement in region 6 is also observed, which corresponds to the amino acids of the flexible loop involved in enzyme catalysis (Figure 7) [23]. Thus, it can be suggested that the disparities in susceptibility to thiol reactive agents between the different proteins produced by changes in regions 1 and 4 are due to the effect of their side chains and not to any major rearrangement of the main chain.

### The pKa of the interface Cys is regulated by regions 1 and 4

In previous work, we reported that a factor that controlled the reactivity of the interface Cys in TIM from T. brucei and T. cruzi is the pKa of its thiol group, which is 0.8 pH units lower in TcTIM than in TbTIM [24]; the respective values were 9.28±0.07 and 10.08±0.03. When the pKa of the thiol group for Cys 14/15 of the wild type enzymes and the chimerical enzyme TcTIM 2,3, 5–8 were determined anew, under the same conditions, the values turned out to be 9.27, 10.53 and 10.61 for TcTIM, TbTIM and TcTIM 2,3, 5–8, respectively (Table 3). Interestingly the interface Cys of TcTIM 1–4 (with a susceptibility to thiol reactive agents like TcTIM) had a pKa of 9.17, and those of chimeras TcTIM 1–3, TcTIM 1 and TcTIM 1–3, 5–8 (with an intermediate susceptibility to thiol reactive reagents) had pKas of 9.86, 9.49 and 9.59, respectively.

**Table 3:** pKa values of Cys 14/15 in TbTIM and TcTIM and some mutant enzymes.

Enzyme	MMTS concentration used (µM)	pKa
TbTIM	80	10.53±0.06
TcTIM	10	9.27±0.16
TcTIM 1–4	10	9.17±0.12
TcTIM 1–3	10 and 80	9.86±0.05
TcTIM 1	80	9.49±0.08
TcTIM 1–3, 5–8	10 and 80	9.59±0.06
TcTIM 2,3, 5–8	80	10.61±0.06

## Discussion

In this work, we have developed a method in which, by taking the same protein from two evolutionary closely related organisms, it is feasible to locate the amino acids responsible for a given property of a protein. It is noteworthy that the method is not biased by structural or hypothetical considerations; it is an experimental approach that indicates the protein region (or protein regions) relevant for a given function.

This strategy is probably best suited for proteins with a high level of homology. For example, our attempts to build chimeras of TcTIM and TIM from Homo sapiens (TcTIM 1–4: HsTIM 5–8 and HsTIM 1–4: TcTIM 5–8) yielded catalytic inert proteins. Most likely, the proteins did not fold correctly, since we observed that, upon expression, the proteins were predominantly found in inclusion bodies. Nonetheless, our experimental approach may be useful in the study of other pairs of proteins from the TIM barrel superfamily or other protein families, particularly if they have highly similar amino acids sequences.

Before beginning this study, it was not possible to predict which mutations would affect the catalytic properties and the susceptibility of the interface Cys to sulfhydryl reagents. However, by grafting different portions of the proteins, we identified two separate and discrete regions of the protein (regions 1 and 4) that establish the resistance/susceptibility of the interface Cys to thiol reagents. Taken as a whole, our data show several points that are noteworthy. First, although some of the chimeras exhibited relatively low kcat, the catalytic efficiency of all the chimeras was comparable to those of the WT enzymes. This is not surprising, since the catalytic amino acids K13, H95 and E167 are strictly conserved and, thus, the exchange of one region for another did not alter them; in consequence catalysis was not affected. Nevertheless, it is somewhat remarkable that the introduction of regions with a significant number of different residues did not affect the catalytic events, indicating that the exchange of regions with different amino acid composition is well tolerated. Second, the properties of the interface Cys are not the result of a gradual and continuous change, indicating that all parts of the protein do not contribute to the properties of the interface Cys and that instead, they are modulated by a small number of amino acids. In fact, substitution of region 5 to 8 which together comprise 32 of the total 65 amino acid differences between the two WT enzymes did not affect the reactivity of the interface Cys. This strongly suggests that a feature of a given residue, or at least that of the interface Cys, is not a global phenomenon, instead it appears to depend on the integrity and communication between a few residues. Third, of the 18 amino acid differences in regions 1 and 4 of WT TcTIM and WT TbTIM, only one of them may be considered part of the dimer interface. This is completely opposite to our original hypothesis, in which we thought that the susceptibility determinants of MMTS action were localized in the interface. In this regard, we note that mutants in which the interfacial residues of TbTIM were incorporated into TcTIM, and vice versa, did not significantly alter the susceptibility of the respective enzymes to MMTS. Fourth, none of the different residues in regions 1 and 4 contact the interface Cys or the residues surrounding it. Thus, the overall data indicate that amino acids of regions that are distant from the interface Cys determine its reactivity to the sulfhydryl reagent (Figure 4). Further studies will be needed to locate more precisely the minimum number of residues involved in MMTS susceptibility.

Our study is an example of how long-range interactions, or perhaps evolutionary protein segments that apparently have no structural coherence [14], can determine the behavior and properties of different parts of the protein in a given milieu. Thus, we would like to point out that our experimental approach, using a modular, systematic approach, and not a random mutagenesis method, together with results that give new insights into the factors that control the properties of protein-inhibitor interactions, could be of value in studies that probe protein-function relationships.

## Materials and Methods

### Design of the genes of chimerical proteins

DNA sequences X03921 and U53867 at the NCBI database for TbTIM and TcTIM, respectively, were used for the design of the three chimerical proteins: TcTIM 1–6, TcTIM 1–5 and TcTIM 1–4. These genes were synthesized by GenScript (Piscataway NJ). The gene for chimera TcTIM 1–4 was planned in such a manner that it could serve as basis for the construction of other chimerical proteins. The sequence of TcTIM 1–4 was slightly altered so that it included a restriction site for HaeII between bases 292 and 300. Using this restriction enzyme, both, regions 4 from TbTIM or TcTIM could be obtained and the chimerical proteins TcTIM 1–3 and TcTIM 4 could be constructed. The gene for chimera TcTIM 1–3, 5–8 was also synthesized by GenScript. Three PCR reactions, using Accuzyme DNA polymerase (Bioline, Taunton MA), were necessary to obtain chimera TcTIM 1–2. The external T7 promoter oligonucleotide and the sequence 5′GCGTTCTGTGCGGCAATCTG3′ (Rv12) were used to amplify regions 1 and 2 of TcTIM using DNA from chimera TcTIM 1–4 as a template. The external T7 terminator oligonucleotide and the sequence 5′CAGAACGCCATTGCAAAGAGC3′(Fw38) were used to amplify regions 3 to 8 from TbTIM using WT TIM DNA as a template.

This same strategy was used to make chimera TcTIM 1 using the same external oligonucleotides and the sequences 5′GTGCAATGCGTAGTGGCCTCC3′(Fw28) and 5′TGATCACGATGTGCAATGCGT3′(Rv1). The template DNAs were from chimera TcTIM1–4 and from TbTIM for the first and second PCR reactions, respectively.

These same oligonucleotides were used to build chimera TcTIM 2,3, 5–8. The DNA of TbTIM was taken as a template with the T7 promoter and sequence Rv1 to amplify region 1 from TbTIM and join it to regions 2–8 from chimera TcTIM 1–3, 5–8 amplified with the T7 terminator and sequence Fw28 using the DNA of this same chimera as a template.

The mutant enzyme TcTIM 2,3, 5–8:19E, 20S, 21L, 23V, 24P was also built using three PCR reactions. The DNA of chimera TcTIM 2,3, 5–8 was used as a template together with the following mutagenic nucleotides: 5′GCTCCGAAAGCCTGTTGGTTCCGCTTATTGATCTGTTTAACTCC3′ (Fw) and 5′CGAGGCTTTCGGACAACCCAGGCGAATAACTAGACAAATTGAGG3′ (Rv).

The mutant enzymes TcTIM 2,3, 5–8:19E, 20S; TcTIM 2,3, 5–8: 21L, 23V and TcTIM 2,3, 5–8: 24P were prepared with the QuikChange site directed mutagenesis kit and the following sequences: for TcTIM 2,3, 5–8:19E, 20S 5′GCAACGGCTCCGAAAGCTCTTTGTCGG3′ (Fw) and 5′CGTTGCCGAGGCTTTCGAGAAACAGCC 3′ (Rv), for TcTIM 2,3, 5–8: 21L, 23V 5′AACGGCTCCCAACAGCTGTTGGTTGAG3′ (Fw) and 5′TTGCCGAGGGTTGTCGACAACCAACTCG3′(Rv), and for TcTIM 2,3, 5–8: 24P 5′GTCGCCGCTTATTGA TCTGTTTAACTCC3′ (Fw) and 5′CAGCGGCGAATAACTAGACAAATTGAGG3′ (Rv), respectively.

All genes of the chimerical proteins were cloned into the pET-3a expression plasmid using the Nde-I and BamHI restriction sites. Every gene was completely sequenced and transformed into BL21(DE3)pLysS cells (Novagen, Madison WI).

### Expression and purification of chimerical proteins

Bacteria containing the plasmids with each of the chimerical genes were grown in Luria Bertani medium supplemented with 100 µg/mL ampicillin and were incubated at 37°C. Once the cell cultures reached an A_600 nm_ = 0.6, a final concentration of 0.4 mM isopropyl -β-D thiogalactopyranoside was used for induction and the bacteria were incubated 12 h more at 30°C before harvesting them.

Since TcTIM and TbTIM have different purification protocols described in references [6] and [25], respectively, some modifications had to be introduced to purify the chimerical proteins containing different regions of each sequence. TcTIM tends to distribute mainly in the soluble fraction of the bacterial extract, while TbTIM tends to be with the membrane fraction and has to be solubilized with 300 mM NaCl. Each chimerical enzyme was subjected to a preliminary test to determine if it was mainly localized in the soluble supernatant or the insoluble fraction. They were then treated accordingly as TcTIM or TbTIM, respectively (Table 1).

After the 12 h induction, bacteria were collected by centrifugation and the cells were resuspended in 40 mL of lysis buffer (100 mM MES, 1 mM DTT, 0.5 mM EDTA and 0.2 mM PMSF, pH 6.3). In the case of those chimerical enzymes that distributed mainly to the insoluble fraction the lysis buffer additionally contained 300 mM NaCl. Each suspension was sonicated 5 times for 40 seconds, with 1 min rest between each cycle. The sonicated suspensions were then centrifuged at 144000× g for one hour to separate the cellular debris from the soluble fraction.

The supernatants of the chimerical enzymes treated like TbTIM were diluted to have a final salt concentration of approximately 20 mM before application to the column. All supernatants were applied to a fast flow SP-sepharose column that had been equilibrated previously with 50 mM MES buffer pH 6.3, and the protein was eluted with a 0–500 mM NaCl gradient in the same buffer. The eluted protein was pooled and precipitated under agitation with 70% (w/v) ammonium sulfate for 12 h. The precipitate was centrifuged at 23000× for 15 min and dissolved in 3 mL of 100 mM triethanolamine (TEA), 1 mM EDTA pH 7.4. To this solution enough ammonium sulfate was added to have a final concentration of 2.2 M and was applied to a hydrophobic Toyopearl column, which had been previously equilibrated with 100 mM TEA, 1 mM EDTA, pH 7.4 and 2.2 M ammonium sulfate. The chimerical proteins were eluted with a linear gradient of ammonium sulfate of 2.2 to 0 M. The fractions containing enzyme were pooled and concentrated to have 1 mg/mL or more of protein concentration. All steps of the different purifications were monitored with SDS-PAGE gels (16% acrylamide) stained with Coomassie Blue and by measuring catalytic activity. All proteins were stored at 4°C in 70% ammonium sulfate at concentrations greater than 1 mg/mL.

At intermediate stages of the purification process of all chimerical enzymes, protein concentrations were determined using the bicinchoninic acid method (BCA, protein assay reagent kit) at 562 nm and the molar extinction coefficients at 280 nm for purified proteins were 36440 M^−1^ cm^−1^ for WT TcTIM, TcTIM 1–6, TcTIM 1–5, TcTIM 1–4, TcTIM 1–3, TcTIM 1–3, 5–8 and TcTIM 2,3, 5–8 and 34950 M^−1^ cm^−1^ for WT TbTIM, TcTIM 4, TcTIM 1–2 and TcTIM 1, respectively.

### Activity assays

Enzyme activity was measured at 25°C following the conversion of glyceraldehyde 3 –phosphate (GAP) to dihydroxyacetone phosphate using α-glycerolphosphate dehydrogenase (α- GDH) as coupling enzyme. NADH oxidation was monitored at 340 nm. The reaction mixture had 100 mM TEA, 10 mM EDTA, pH 7.4, 1 mM GAP, 0.2 mM NADH and 20 µg/mL α-GDH. The reaction was initiated by addition of 5 ng/mL of the corresponding TIM. To calculate the kinetic parameters, GAP concentration was varied between 0.05 and 2 mM. The data were adjusted to the model of Michaelis and Menten and the values of *K*m and Vmax were calculated by non-linear regression.

### Inactivation assays with MMTS

Both, WT enzymes, as well as chimerical enzymes, at a concentration of 250 µg/mL were incubated with the indicated concentrations of MMTS in a buffer containing 100 mM TEA, 10 mM EDTA, pH 7.4 for 2 h at 25°C. At this time the mixtures were diluted and an aliquot of the dilution was withdrawn to measure activity at a concentration of 5 ng/ml of reaction mixture. The activity data are reported as percentage of residual activity, taking the activity of each corresponding enzyme in the absence of MMTS as 100%.

### Number of Cys derivatized by DTNB as a function of time

The number of Cys derivatized by DTNB was determined for WT TbTIM, WT TcTIM and 9 chimeras (Table 2) essentially as described in reference [11]. In this case, all the enzymes (200 µg) were incubated in at 25°C in 1 mL of a buffer containing 100 mM TEA, 10 mM EDTA and 1 mM DTNB pH 7.4 for 20 min. The absorbance at 412 nm was recorded immediately after adding the enzymes. The value of a blank, with no enzyme, was subtracted from the experimental values. The number of derivatized Cys at 8 and 12 min was calculated with the equation
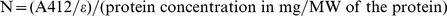
where A is the absorbance and ε is the extinction coefficient of nitrobenzoic acid, 13,600 M^−1^ cm^−1^ .

### Determination of the pKa of the interface Cys

The pKa of the interface Cys of WT TbTIM, WT Tc TIM and of 5 chimeras, including TcTIM 2,3, 5–8 (Table 3), was determined as described in reference [24] with some modifications. Briefly, the enzymes were incubated at a concentration of 5 µg/mL in 100 mM TEA and 10 mM EDTA adjusted to the desired pH; MMTS at a concentration of 10 or 80 µM was also added. For chimeras that had an MMTS-inactivation profile similar to TbTIM we used 80 µM MMTS and for those with a profile similar to TcTIM we used 10 µM MMTS. For chimeras with an intermediate MMTS-inactivation profile, both concentrations were used and the mean of the result of both experiments was taken as the pKa value. The apparent pKa of the interface Cys was determined from plots of ln of percent remaining activity versus pH. The data were fitted to a model derived from the Henderson-Hasselbach equation:
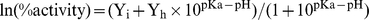
where Y_i_ and Y_h_ represent the initial and final activities, respectively.

The rest of the procedure and the conditions and corrections applied were performed as previously described [24].

### Crystallization of chimera TcTIM 2,3, 5–8 and data collection

Chimera TcTIM 2,3, 5–8 was crystallized via vapor diffusion using the sitting drop method. One µl of a solution of protein at 35 mg/ml was mixed with 1 µl of reservoir solution. Crystals were obtained in the H3 condition of the Index HT kit (Hampton Research) after 1 or 2 weeks of incubation. The best crystals were grown at 9°C and obtained with a reservoir solution of 200 mM sodium malonate and 20% polyethylene glycol 3350. The crystals were cryoprotected by increasing the concentration of polyethylene glycol 3350 in the crystal drop to 35% and they were immediately frozen in liquid nitrogen. Diffraction data were collected at the Life Sciences Collaborative Access Team (LS-CAT) 21-ID-F beamline in the Advanced Photon Source (Argonne National Laboratory), using a MarMosaic 225 detector. The data were processed with MOSFLM [26] and reduced with SCALA [27].

### Structure Determination and Refinement

The structure was solved by the molecular replacement method with the program PHASER [28] using the coordinates of the native TcTIM at 1.8 Å resolution (Protein Data Bank code 1TCD) as the search model. Refinement was made with the programs Refmac [29] and Phenix [30], followed by model building with COOT [29]. The existence of the mutations in regions 1 and 4 was initially confirmed by difference Fourier maps calculated using the structure of TcTIM. On two of the four monomers in the asymmetric unit, residues 170–177 (loop 6 or flexible loop) are less clear on the electron density map. These regions are normally poorly defined in apo-TIMs. Five percent of the data were used to validate the refinement. σA-weighted, F_o_ - F_c_ simulated annealing omit maps were used to further validate the quality of the model. Data collection and refinement statistics are given in Table 4. Figures were generated with PyMOL (available on http://www.pymol.org/). The atomic coordinates and structure factors (code 3Q37) have been deposited in the Protein Data Bank, Research Collaboratory for Structural Bioinformatics, Rutgers University, New Brunswick, NJ (http://www.rcsb.org/).

**Table 4:** X-ray data collection and refinement statistics, Values in parentheses are for the last resolution shell.

Parameters	
Data collection statistics	
Space group	P2_1_
Unit cell dimensions	
a, b, c (Å)	83.6, 77.2, 85.4
α, β, γ angles (degrees)	90.0, 116.6, 90.0
Resolution range (Å)	54.3-1.65 (1.74-1.65)
Number of reflections	336,767 (40,524)
Number of unique reflections	107,171 (13,903)
Data completeness (%)	91.9 (82.4)
R_sym_ (%)^a^	7.3 (27.5)
I/σ	6.9 (2.3)
Mn(I)/sd	10.5 (3.9)

a R_sym_ is defined as ∑|(I-<I>)|/∑I, where I is the intensity individual reflection and <I> is the average intensity for this reflection; the summation is over all intensities.

b R_cryst_ = |F_o_|−∥F_c_|/F_o_ for all reflections.

c R_free_ is the same as R_cryst_, but calculated on the 5% of data excluded from refinement.

d No σ cut-offs used on the refinement (F>0σF).

e Engh RA, Huber R (1991) Acta Cryst A47: 392–400. Engh RA, Huber R (2001) International Tables for Crystallography, Vol. F, edited by M. G. Rossmann & E. Arnold, pp. 382–392. Dordrecht: Kluwer Academic Publishers.

f Kleywegt GJ, Jones TA (1996). Structure 15: 1395–1400.

## Supporting Information

Figure S1 **Effect of MMTS on WT TcTIM, WT TbTIM and on mutants TcTIM 2,3, 5–8: 19E, 20S; TcTIM 2,3, 5–8: 21L 23V and TcTIM 2,3, 5–8: 24P.** The enzymes were incubated at a concentration of 250 µg/mL in 100 mM TEA, 10 mM EDTA, and the indicated concentrations of MMTS (pH 7.4) for 2 h. At that time the activity of the samples was determined, including a sample without MMTS to calculate the percentage of remaining activity.
(TIF)

Table S1 **Kinetic constants of mutants TcTIM 2,3 5–8: 19E, 20S, 21L, 23V, 24P; TcTIM 2,3, 5–8: 19E, 20S; TcTIM 2,3, 5–8: 21L 23V and TcTIM 2,3, 5–8: 24P.**
(DOC)
