# Supplementary figures and images for: Identification of Amino Acids that Account for Long-Range Interactions in Two Triosephosphate Isomerases from Pathogenic Trypanosomes
Source: PLoS One. 2011 Apr 18;6(4):e18791. doi: 10.1371/journal.pone.0018791 (PMC3078909; doi:10.1371/journal.pone.0018791)

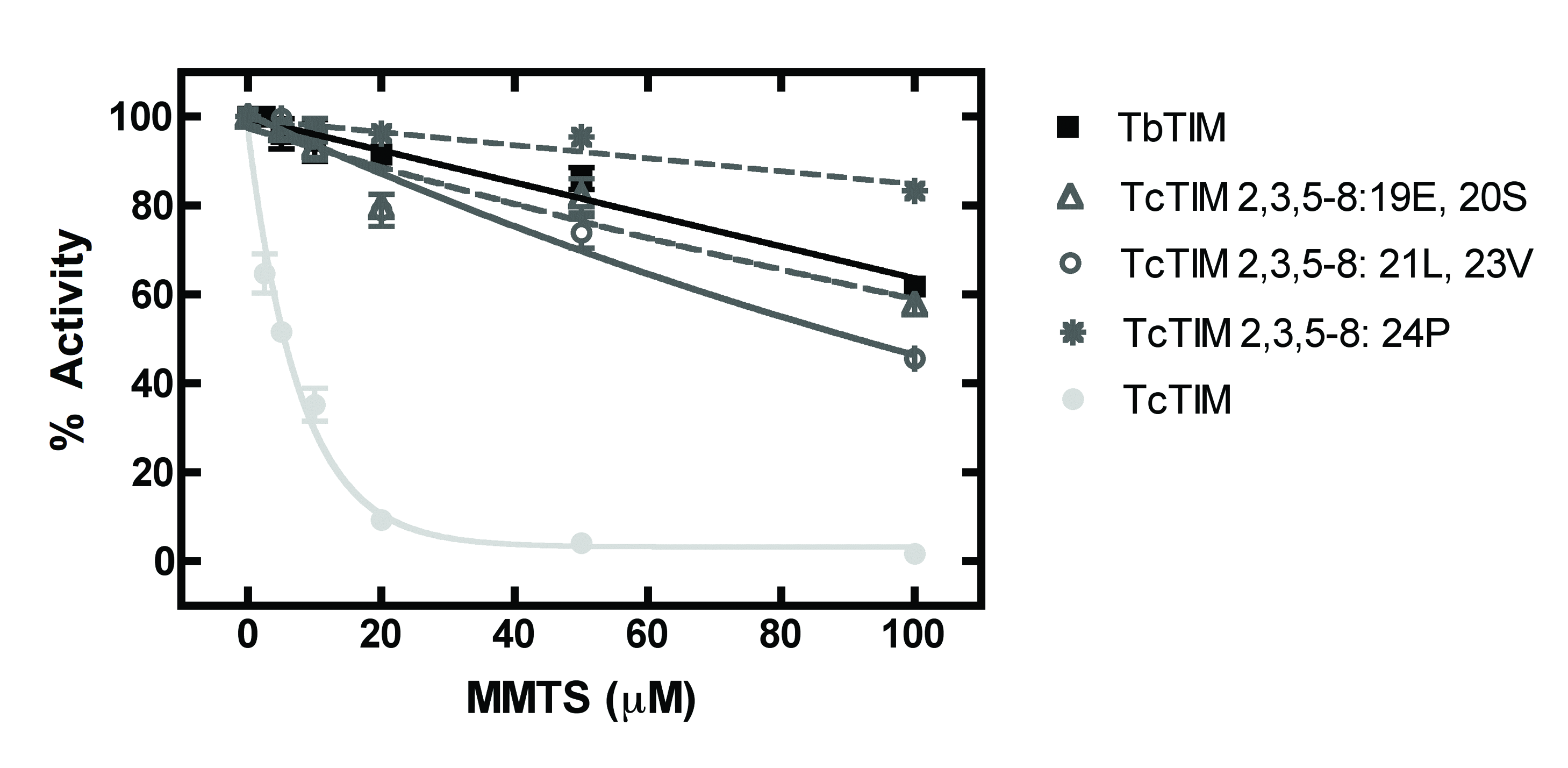

Supplement: Figure S1 — Effect of MMTS on WT TcTIM, WT TbTIM and on mutants TcTIM 2,3, 5–8: 19E, 20S; TcTIM 2,3, 5–8: 21L 23V and TcTIM 2,3, 5–8: 24P. The enzymes were incubated at a concentration of 250 µg/mL in 100 mM TEA, 10 mM EDTA, and the indicated concentrations of MMTS (pH 7.4) for 2 h. At that time the activity of the samples was determined, including a sample without MMTS to calculate the percentage of remaining activity. (TIF) [file pone.0018791.s001.tif]
