# Supplementary material for: Identification of Amino Acids that Account for Long-Range Interactions in Two Triosephosphate Isomerases from Pathogenic Trypanosomes
Source: PLoS One. 2011 Apr 18;6(4):e18791. doi: 10.1371/journal.pone.0018791 (PMC3078909; doi:10.1371/journal.pone.0018791)
Supplement: Table S1 — Kinetic constants of mutants TcTIM 2,3 5–8: 19E, 20S, 21L, 23V, 24P; TcTIM 2,3, 5–8: 19E, 20S; TcTIM 2,3, 5–8: 21L 23V and TcTIM 2,3, 5–8: 24P. (DOC) [file pone.0018791.s002.doc]

| Enzyme | *K*m (mM) | *k*cat ×10^5^ (min^-1^) | *k*cat/*K*m × 10^7^(M^-1^ s^-1^) |
| --- | --- | --- | --- |
| TcTIM 2,3 5-8: 19E, 20S, 21L, 23V, 24P | 0.48 | 3.41 | 1.17 |
| TcTIM 2,3, 5-8: 19E, 20S | 0.47 | 3.36 | 1.19 |
| TcTIM 2,3, 5-8: 21L 23V | 0.51 | 4.15 | 1.35 |
| TcTIM 2,3, 5-8: 24P | 0.22 | 2.55 | 1.85 |
